# Outcomes of carbon ion radiotherapy compared with segmentectomy for ground glass opacity-dominant early-stage lung cancer

**DOI:** 10.1186/s13014-023-02387-1

**Published:** 2023-12-18

**Authors:** Teruaki Mizobuchi, Akihiro Nomoto, Hironobu Wada, Naoyoshi Yamamoto, Mio Nakajima, Takehiko Fujisawa, Hidemi Suzuki, Ichiro Yoshino

**Affiliations:** 1 Department of General Thoracic Surgery, Social Welfare Organization Saiseikai Imperial Gift Foundation, Chibaken Saiseikai Narashino Hospital, 1-8-1 Izumi-Cho, Narashino-Shi, Chiba, 275-8580 Japan; 2 Department of Radiology, Teikyo University School of Medicine, Tokyo, Japan; 3 Department of Pulmonary Surgery, International University of Health and Welfare Narita Hospital, Chiba, Japan; 4 Department of Internal Medicine, Chosei Municipal Hospital, Chiba, Japan; 5 National Institutes for Quantum Science and Technology QST Hospital, Chiba, Japan; 6 Chiba Foundation for Health Promotion and Disease Prevention, Chiba, Japan; 7 Departments of General Thoracic Surgery, Departments of General Thoracic Surgery, Graduate School of Medicine, Chiba University, Chiba, Japan

**Keywords:** Lung cancer, Carbon ion radiotherapy, Segmentectomy, Ground glass opacity

## Abstract

**Purpose:**

This study aimed to compare the outcomes of patients with ground-grass opacity (GGO)-dominant non-small cell lung cancer (NSCLC) who were treated with carbon ion radiotherapy (CIRT) versus segmentectomy.

**Methods:**

A retrospective review of medical records was conducted. The study included 123 cases of clinical stage 0/IA peripheral NSCLC treated with single-fraction CIRT from 2003 to 2012, 14 of which were determined to be GGO-dominant and were assigned to CIRT group. As a control, 48 consecutive patients who underwent segmentectomy for peripheral GGO-dominant clinical stage IA NSCLC were assigned to segmentectomy group.

**Results:**

The patients in CIRT group, compared with segmentectomy group, were significantly older (75 ± 7.2 vs. 65 ± 8.2 years, *P* = 0.000660), more likely to be male (13/14 vs. 22/48, *P* = 0.00179), and had a lower forced vital capacity (91 ± 19% vs. 110 ± 13%, *P* = 0.0173). There was a significant difference in the 5-years overall survival rate (86% vs. 96%, *P* = 0.000860), but not in the 5-years disease-specific survival rate (93% vs. 98%, *P* = 0.368).

**Discussion:**

Compared with segmentectomy, CIRT may be an alternative option for patients with early GGO-dominant NSCLC who are poor candidates for, or who refuse, surgery.

## Introduction

Lung cancer is the leading cause of cancer morbidity and mortality in men, whereas in women, it ranks third in incidence, after breast and colorectal cancer, and second in mortality, after breast cancer [1]. Surgery is the gold standard treatment for early-stage lung cancer [2]. A Japanese lung cancer registry study of 18,973 lung cancer patients treated with surgery in 2010 revealed 5-years overall survival (OS) rates of 97.0%, 91.6%, 81.4%, 74.8%, 71.5%, 60.2% and 58.1% for clinical stages 0, IA1, IA2, IA3, IB, IIA, and IIB disease according to the TNM classification of malignant tumors, eighth edition, respectively [3]; these rates indicate improvements compared with previous reports [3–5].

Although lobectomy has long been the standard surgical procedure for early-stage lung cancer [6], the efficacy of sublobar resection, such as segmentectomy or partial lung resection, has been explored by various investigators. The results of the 1995 Lung Cancer Study Group randomized clinical trial of surgical reduction for lung cancer showed a three-fold increase in the local recurrence rate and a higher mortality rate in the sublobar resection group compared with the lobectomy group [7]. As a result, the sublobar resection became a passive procedure for patients who cannot tolerate lobectomy, e.g., those with low lung function or other comorbidities.

Around the same era, carbon ion radiotherapy (CIRT) was proposed as an alternative to lung resection for lung cancer patients who were deemed inappropriate for, or refused to undergo, surgery. The first clinical trials of CIRT for non-small cell lung cancer (NSCLC) were initiated at the National Institutes for Quantum Science and Technology QST hospital in June 1994 [8–16]. For peripheral stage I lung cancer, the number of radiation fractions and treatment period were reduced from 18 fractions over 6 weeks to 9 fractions over 3 weeks, and then further to 4 fractions over 1 week, respectively, while maintaining safety and efficacy. Based on the results of those clinical trials, a phase I/II study (protocol 0201) was performed in which a dose escalation method was used to determine the optimal dose over a 9-years period, from April 2003 to February 2012 [17]. The initial treatment dose was 28 Gy (relative biological effectiveness [RBE]) administered in a single fraction using respiratory-gated and four-portal oblique irradiation directions, with the total dose escalated to a maximum of 50 Gy (RBE) at increments of 2.0 Gy (RBE). In 123 cases of clinical stage T1N0M0 NSCLC, the 3-years local control rates after irradiation with 28–34 Gy (RBE), 36–42 Gy (RBE), and 44–50 Gy (RBE) were 80.7%, 88.0%, and 90.8%, respectively. Accordingly, we concluded that single-fraction CIRT for clinical stage T1N0M0 NSCLC obtained excellent results, comparable with those of previous fractionated regimens. Therefore, in this cohort, 14 NSCLC cases determined to be GGO-dominant with a GGO diameter to maximum tumor diameter ratio ≥ 50%, according to high-resolution computed tomography (CT), were selected and included in the CIRT group.

The Japan Clinical Oncology Group (JCOG) reported in the JCOG0201 trial that among NSCLC with a maximum tumor diameter ≤ 2 cm, those with extensive GGO on chest CT are pathologically noninvasive [18]. Based on those results, the JCOG0802/WJOG4507L randomized phase III clinical trial was conducted to compare lobectomy with segmentectomy for peripheral NSCLC with a maximum tumor diameter ≤ 2 cm and a maximum diameter of the largest consolidation to maximum tumor diameter ratio > 0.5 [19]. The 5-years overall survival rate was better after segmentectomy than after lobectomy at a median follow-up of 7.3 years (94.3% vs. 91.1%). We obtained significant evidence-based results suggesting that segmentectomy is an acceptable option for peripheral NSCLC with an overall tumor diameter ≤ 2 cm, including tumors with GGO. At the time of this clinical trial, the Department of General Thoracic Surgery, Chiba University Hospital began to perform more aggressive segmentectomies, which led to a more standardized technique. Therefore, 48 consecutive patients who underwent segmentectomy at Chiba University Hospital from 2008 to 2015 for peripheral GGO-dominant clinical stage IA NSCLC were included in segmentectomy group. To our knowledge, this is the first study to compare the outcomes of patients with GGO-dominant NSCLC who were treated with CIRT versus segmentectomy.

## Patients and methods

### Patients

In a phase I/II study (protocol 0201) conducted at the National Institutes for Quantum Science and Technology QST hospital from April 2003 to February 2012, the optimal CIRT dose was determined using a dose escalation method [17]. The initial treatment dose was 28 Gy (RBE) administered in a single fraction using respiratory-gated and four-portal oblique irradiation directions, with the total dose escalated to a maximum of 50 Gy (RBE) in 2.0 Gy (RBE) increments. Single-fraction CIRT was applied to 123 clinical stage 0/IA (TNM classification of malignant tumors, eighth edition) peripheral NSCLC, of which 14 were determined to be GGO-dominant by high-resolution CT and were included in CIRT group of this study.

We have participated in several sublobar surgery clinical trials for lung cancer [19–21] and performed high-quality lung sublobar surgeries, which have undergone internal and external reviews and continue to undergo quality assurance. During those clinical trials, the Department of General Thoracic Surgery, Chiba University Hospital began to perform more aggressive segmentectomies, which led to a more standardized technique. Therefore, 48 consecutive patients who underwent segmentectomy at Chiba University Hospital from 2008 to 2015 for peripheral GGO-dominant clinical stage IA NSCLC were included in segmentectomy group of this study.

The study has been approved by the institutional ethical committees of both Chibaken Saiseikai Narashino Hospital (approval number: 2019–12) and Chiba University (approval number: 3350). This study complied with the protocol, the current version of the Declaration of Helsinki. Accordingly, the medical records of all 62 patients were reviewed and analyzed retrospectively according to the approved protocol.

### Administration of CIRT

A single carbon-ion beam treatment using the four-dimensional radiotherapy (4DRT) technique was performed for clinical stage I peripheral non-small cell lung cancer [17]. Briefly, carbon ion beams (290, 350, and 400 MeV) generated by the Heavy Ion Medical Accelerator at the Chiba Synchrotron were shaped three-dimensionally to fit the tumor contour. A diffuse Bragg peak (SOBP) ensured dose coverage with the center of the SOBP as the reference point; the HIPLAN system was used for CT planning, and respiratory-gated CT images were used. A fixation device was used for patient positioning, and respiratory-gated irradiation was used to minimize tumor movement. A margin of 10 mm was taken from the gross tumor volume, including the spinous process and pleural indentation, where possible, as the clinical target volume (CTV). The internal margin (IM) corresponded to the movement of the target during gating, and the planned target volume (PTV) was the CTV plus IM [7]. The carbon ion dose was expressed in Gy (RBE), calculated by multiplying the physical dose by the relative biological effect (RBE), approximately 3.0 at 0.8 cm from the distal end of the SOBP.

CIRT was performed within 1 week after treatment planning. The 14 patients were prescribed doses of 32.0–46.0 GyE in 1 fraction (Protocol #0201) (Table 1). Toxicity to organs such as the lung parenchyma, lung hilum, parietal pleura, and skin was assessed according to the Radiation Therapy Oncology Group/European Organization for Research and Treatment of Cancer criteria [22]. Given the increased risk of radiation-induced pneumonitis in pulmonary carbon-ion radiotherapy, caution should be exercised when employing carbon-ion radiotherapy for non-small cell lung cancer without utilizing 4DRT, which is our main concept.

**Table 1:** Demographic characteristics of patients in CIRT and segmentectomy groups before treatment

	CIRT group (n = 14)	Segmentectomy group (n = 48)	*P* value
Mean age + SD (year-old)	75 ± 7.2	65 ± 7.2	0.000660
Mean Body Mass Index ± SD (kg/m^2^)	22 + 2 1	23 + 3.3	0.366
Gender: Male/female	13/1	22/26	0.00179
ECOG Performance Status: 0/1	10/4	40/8	0.321
Charlson comorbidity index			0.593
Low: 0	3	18	
Medium: 1–2	6	17	
High: 34	4	12	
Very high: ≥ 5	1	1	
Mean maximum tumor size ± SD (mm)	25 + 8.6	17 + 4.7	0.00543
Mean maximum consolidation size + SD (mm)	4.7 ± 5.5	4.2 ± 3.6	0.738
Mean consolidation/tumor size ± SD	0.17 ± 0.16	0.23 + 0.19	0.245
Clinical stage (UICC8)			0.773
TlisNOMO	2	12	
UmiNOMO	6	19	
TlaNOMO	5	15	
TlbNOMO	0	1	
TIcNOMO	1	1	
Histology			N/A
Adenocarcinoma	14	48	
Non-adenocarcinoma	0	0	
Spirometry			
Mean %FVC ± SD (%)	91 ± 19	106 + 13	0.0173
Mean %FEV1 ± SD (%)	103 + 27	103 + 14	0.965
Mean FEV1% ± SD (%)	77 + 15	77 ± 6.3	0.996
Mean smoking Index + SD (pack-year)	38 + 41	19 + 26	0.146

### Follow up

The first follow-up examination was performed 4 weeks after CIRT and included a physical examination, blood chemistry analysis, and CT. Subsequent follow-up was performed every 3–4 months. If recurrence was suspected, 18F-fluorodeoxyglucose positron emission tomography (FDG-PET) was performed for confirmation. Each clinical finding was identified based on a consensus among the research board members. Because it was difficult to differentiate normal tissue responses to radiation from tumor regrowth, temporarily enlarged densities seen approximately 3 months after CIRT were considered locally controlled tumors. The following findings were defined as local recurrence: a gradual increase in tumor size on follow-up CT or an increase in tracer uptake on FDG-PET, increasing levels of tumor markers, and, if applicable, identification of the recurrence was performed by endobronchial biopsy in the tumor and by endobronchial ultrasound-guided transbronchial needle aspiration in the hilar and mediastinal lymphadenopathy [23]. In segmentectomy group, according to the National Comprehensive Cancer Network guidelines [2], outpatient follow-up was performed every 3 months similarly to CIRT group.

### Statistical analysis

Statistical analysis was performed using the StatMate V (version 5.01) software package (Nihon 3B Scientific Inc., Niigata, Japan), abiding by the statistical and data reporting guidelines [24]. Means, standard deviations, medians, and ranges were calculated for continuous variables, and percentages for categorical variables at baseline. The equal-variance two-sample t-test and chi-square test were used to compare patient demographic and clinical characteristics at baseline between the two treatment groups (CIRT versus surgery). All continuous variables, except the median follow-up period after treatment, are expressed as means ± standard deviation. Spirometric data, expressed as continuous variables, were evaluated using the paired *t*-test. Survival analysis was performed using the Kaplan–Meier method [25]. Survival probabilities were compared by the log-rank test. *P* < 0.05 was considered statistically significant.

## Results

### Demographic characteristics of patients in CIRT and segmentectomy groups before treatment

The participants included 13 men and 1 woman in CIRT group and 26 men and 22 women in segmentectomy group. The patients in CIRT group, compared with segmentectomy group, had a significantly older age (75 ± 7.2 vs. 65 ± 8.2 years, *P* = 0.000660), higher proportion of males (93% vs. 46%, *P* = 0.00179), greater maximum tumor diameter (25 ± 8.6 vs. 17 ± 4.7 mm, *P* = 0.00543), and lower percentage forced vital capacity (%FVC; 91 ± 19% vs. 110 ± 13%, *P* = 0.0173). On the other hand, there were no differences between the groups in terms of body mass index, Eastern Cooperative Oncology Group performance status (PS), Charlson comorbidity index, maximum consolidation diameter, clinical stage (TNM classification of malignant tumors, eighth edition), histologic type, clinical stage, percentage forced expiratory volume in 1 (%FEV1), the ratio of FEV1 to FVC (FEV1/FVC, also known as FEV1%) and smoking index (Table 1).

### Reasons for undergoing CIRT in CIRT group and CIRT details and results

The decision to use CIRT rather than surgery in the patients in CIRT group was determined as described below. The decision on whether or not a patient was tolerant to surgery was made by our cancer board, consisting of thoracic surgeons, respiratory medicine physicians, and radiotherapy specialists. Case #1 in CIRT group (CIRT#1) was being treated for a second primary lung cancer in the S9 lower lobe of the left lung after bi-lobectomy of the middle and lower lobes of the right lung, and the patient was determined to be intolerant to additional surgery due to poor pulmonary function. Similarly, CIRT#5 was being treated for a second primary lung cancer in S1 + 2 of the left upper lobe after right upper lobectomy, and the patient was considered to be intolerant to surgery due to poor pulmonary function. CIRT#4 was judged to be inoperable due to advanced age and previous lung cancer surgery. CIRT#7 was judged to be operable; however, the patient had end-stage renal failure and refused to undergo surgery. Of the 14 cases, 9, including CIRT#7, were judged to be operable, but the patients refused surgery, and thus CIRT was selected.

The tumor diameter, consolidation diameter/tumor diameter ratio (C/T ratio), CIRT dose, treatment-related complications of grade ≥ 2 [22], post-treatment observation period, and post-treatment results for each patient in CIRT group are shown in Table 2. After CIRT, one patient died of lung cancer due to local recurrence and distant metastasis after 29 months of treatment. On the other hand, 9 of 14 patients died of diseases other than the targeted lung cancer at 80 ± 39 months, and details of the cause of death are provided in Table 2. Four patients did not have recurrence, but new GGOs were detected in one patient by follow-up CT.

**Table 2:** Reasons for undergoing CIRTin CIRTgroup and CIRT details and results

	Reasons for CIRT	Tumor size (mm)	C/T ratio	CIRT dose Gy(RBE)	Complication(> 2)	Follow-up (Months)	Outcomes
CIRT#1	LPH and PL	18	0.11	32		29	DC
CIRT#2	LHF	19	0.47	36	–	35	DO (AMI)
CIRT#3	LPF	44	0.50	38	–	67	DO (TR)
CIRT#4	CJ	28	0.089	40	–	70	DO (TAA)
CIRT#5	LPH and PL	22	0.27	42	–	180	AR
CIRT#6	RS	32	0.13	44	–	94	DO (MOF)
CIRT#7	RS	18	0.22	40	–	148	DO (RF)
CIRT#8	RS	21	0.00	44	–	148	AR
CIRT#9	RS	43	0.15	46	–	110	DO (Pneumonia)
CIRT#10	RS	20	0.050	46	–	66	DO (lleus)
CIRT#11	RS	22	0.023	46	–	150	AR
CIRT#12	RS	25	0.080	46	–	140	AR
CIRT#13	RS	22	0.30	48	–	130	DO (Senility)
CIRT#14	RS	16	0.00	48	–	110	DO (Senility)

### Reasons for undergoing segmentectomy in segmentectomy group

The reasons for undergoing segmentectomy in segmentectomy group were as follows: (1) maximum tumor diameter < 3 cm, (2) tumor localization in the periphery (outer one-third field of the lungs), and (3) GGO-dominant cancer (C/T ratio < 0.5). Segmentectomy was indicated in 43 of 48 cases based on these criteria. On the other hand, 5 of 48 patients underwent segmental resection for other reasons such as comorbidities and/or inability to tolerate lobectomy; the details of these selected cases are shown in Table 3.

**Table 3:** Reasons for undergoing segmentectomy as a passive limited resection and the outcomes

	Reasons for segmentectomy	Age/gender	Tumor size (mm)	C/T ratio	Follow up (Months;	Outcomes
Surgery#15 Surgery#21 Surgery#23 Surgery#24 Surgery#41	Thvmoma, MG LC, LPF CCI, CUP RF, AAA RRTA	62/Female	15	0.00	120	AR
		60/male	18	0.28	130	AR
		62/male	16	0.31	100	AR
		60/female	26	0.19	31	AR
		69/female	11	0.45	62	AR

*AAA* Abdominal Aortic Aneurysm, *AR* Alive without Recurrence, *CCI* Cervical Cord Injury, *CUP* Cancer of Unknown Primary, *MG* Myasthenia Gravis, *LC* Laryngeal Cancer, *LPF* Low Pulmonary Function, *RF* Renal Failure, *RP* Rheumatic Polymyositis, *TA* Takayasu’s Arteritis

### A decline in spirometric parameters after CIRT

Spirometric examination of the 14 patients before CIRT showed the following results: %FVC of 92 ± 20%, %FEV1 of 105 ± 28%, and percentage diffusing capacity of the lungs for carbon monoxide (%DLCO) of 88 ± 28%. Approximately 1 year after CIRT, the spirometric evaluation revealed a mean %FVC of 92 ± 22%, %FEV1 of 104 ± 26%, and %DLCO of 82 ± 34%. The mean rates of change from before to after CIRT in the %FVC, %FEV1, and %DLCO were + 0.11 ± 12% (*P* = 0.989), − 0.90 ± 9.1% (*P* = 0.933), and − 6.1 ± 18% (*P* = 0.619), respectively. Respiratory function tests at approximately 1 year after CIRT showed no statistically significant differences (Table 4), but there were large individual differences, which will require careful follow-up.

**Table 4:** A decline in spire-metric parameters after CIRT

	Before CIRT	After CIRT	Changing rate	*P* value
%FVC(%)	92 + 20	92 + 22	0.11 + 12 ∆	0.989
%FEV1(%)	105 + 28	104 + 26	0.90 + 9.1 ▼	0.933
%DLCO(%)	88 + 28	82 + 34	6.1 + 18 ▼	0.619

*CIRT* Carbon Ion Radio-Therapy, *DLCO* Diffusing Capacity of Carbon Monoxide, *FEV1* Forced Expiratory Volume in One Second, *FVC* Forced Vital Capacity

### Comparison of the 5-years overall survival and disease-specific survival between CIRT and segmentectomy groups

**Fig. 1:**
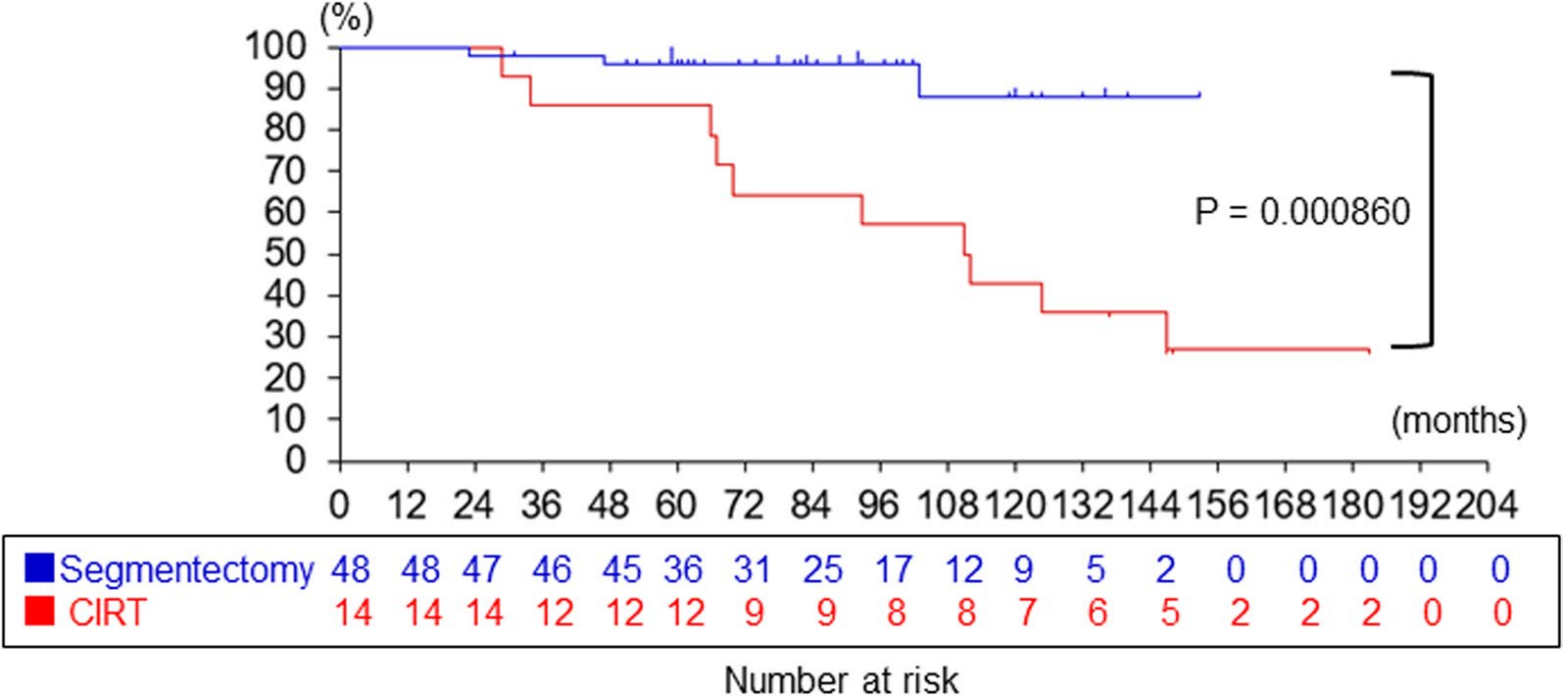
Comparison of overall survival after segmentectomy versus CIRT for lung cancer. Kaplan–Meier estimates of the survival probability at 2.5, 5, and 7.5 years after segmentectomy in 48 patients were 98%, 96%, and 96%, respectively (blue line), and those after CIRT in 14 patients were 93%, 86%, and 64%, respectively (red line). The log-rank test showed inferior survival in CIRT group compared with segmentectomy group (*P* = 0.000860)

**Fig. 2:**
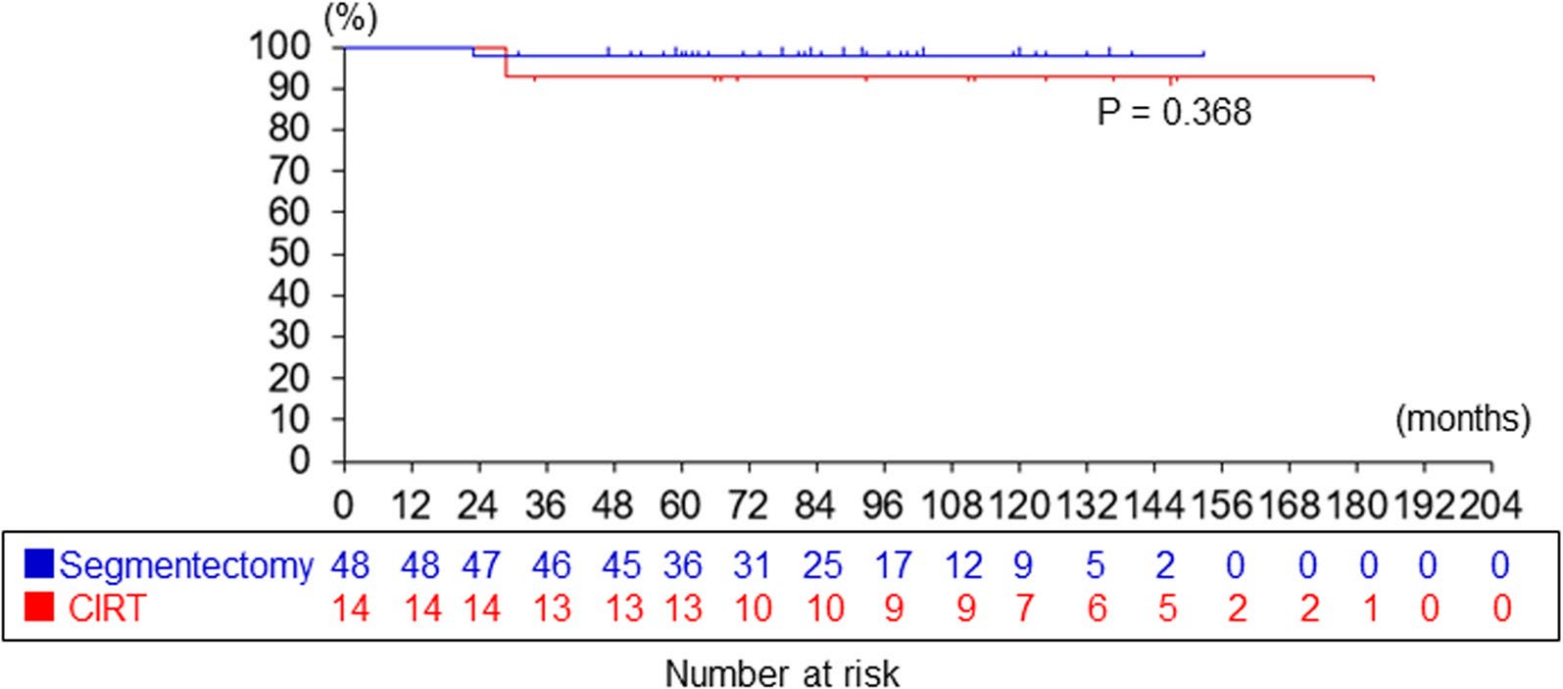
Comparison of disease-specific survival after segmentectomy versus CIRT for lung cancer. Kaplan–Meier estimates of the survival probability at 2.5, 5, and 7.5 years after segmentectomy in 48 patients were 98%, 98%, and 98%, respectively (blue line), and those after CIRT in 14 patients were 93%, 93%, and 93%, respectively (blue line). The log-rank test showed non-inferior survival in CIRT group compared with segmentectomy group (*P* = 0.368)

The median follow-up period was 110 (range, 12–180) months in CIRT group and 84 (range, 6.7–154) months in segmentectomy group. The Kaplan–Meier estimate of overall survival was significantly lower after CIRT than after segmentectomy (86% vs. 96%, *P* = 0.000860; Fig. 1); however, the Kaplan–Meier estimate of disease-specific survival was not statistically different between CIRT and segmentectomy groups (93% vs. 98%, *P* = 0.368; Fig. 2).

## Discussion

Radiotherapy is the primary treatment for medically inoperable patients with early-stage NSCLC, and CIRT is a promising treatment for medically inoperable patients with localized NSCLC because its excellent dose localization allows intensive irradiation of the target while sparing surrounding healthy tissue. Grutters et al. performed a meta-analysis to compare photons, protons, and carbon ions in radiotherapy for NSCLC. They reported adjusted pooled estimates of 2- and 5-years overall survival rates after CIRT for stage I inoperable NSCLC of 74% and 42%, respectively, significantly higher than those for conventional radiotherapy [26]. CIRT with utilizing 4DRT appears to substantially improve the prognosis of early-stage lung cancer compared with conventional radiotherapy. Accordingly, we have reported that CIRT is superior to SBRT and proton beam in therapeutic efficacy and fewer adverse events because CIRT offers better dose distribution and less damage to the normal lung [27].

The population of CIRT group in the present study was obtained from CIRT protocol #0201, a single-fraction dose-escalation clinical study that started in April 2003. In that trial, the total dose was increased from 28 to 50 Gy (RBE). The resulting 3-year local control rates were 80.7%, 88.0%, and 90.8% after treatment with 28–34 Gy (RBE), 36–42 Gy (RBE), and 44–50 Gy (RBE) for stage T1 NSCLC, respectively. Of these doses, 44–50 Gy (RBE) achieved the best results, with no significant adverse reactions [17]. The only case of recurrence in CIRT group (CIRT#1) that occurred in this study was likely due to the low irradiation dose of 32 Gy (RBE), the lowest of the 14 cases.

An analysis conducted using the National Cancer Database in the United States compared the prognoses of 10,032 cases of partial resection and 4296 cases of stereotactic radiotherapy (SBRT) for lung cancer and revealed comparable survival between partial resection with positive margins and SBRT [28]. However, complete lung resection had a lower risk of death compared with SBRT [28]. When we perform segmentectomy resection, the surgical margins are determined according to the report of Sawabata et al.; i.e., a margin of at least the tumor diameter length, or 2 cm, is necessary to prevent marginal recurrence [29]. On the other hand, CIRT is indicated for early-stage lung cancer, especially in patients deemed operable but who refuse surgery, and a sufficient irradiation dose and irradiation coverage should be applied.

The JCOG0802/WJOG4507L trial conducted by Saji et al. revealed surprising results. In particular, overall survival was better in the segmentectomy group than in the lobectomy group despite the high incidence of local recurrence. This was thought to be due to the difficulty in achieving a cure for second cancers after lobectomy [19]. In CIRT group in this study, one of the patients (CIRT#1) was judged to be intolerable to surgery due to low pulmonary function after bi-lobectomy for lung cancer and was consequently assigned to CIRT treatment. If CIRT provides adequate local control of a second lung cancer in patients who have already undergone lobectomy for lung cancer, it may be a curative treatment option. In addition, completion lobectomy may be required for local recurrence following segmentectomy. Because of the high degree of adhesion and extraordinary difficulty in dissecting pulmonary arteries, completion pneumonectomy must be selected in some cases. In our surgery group, local recurrence occurred in case #38 in the residual right upper lobe at 7 years and 1 month after right S3 segmentectomy, and right completion lobectomy was required. Surgery for local recurrence after segmentectomy is very difficult, but if CIRT is effective in such cases, it may become an alternative option.

In conclusion, CIRT group had a significantly older age, more men, lower forced vital capacity in spirometry, and a larger maximum tumor size, but no significant difference in 5-years disease-specific survival compared with segmentectomy group, which predominantly comprised patients with the aggressive segmentectomy criterion. Compared with segmentectomy, CIRT may be an alternative option for patients with early GGO-dominant NSCLC who are poor candidates for, or who refuse, surgery.

## Abbreviations

CIRT: Carbon ion radiotherapy
C/T ratio: Consolidation diameter/tumor diameter ratio
CT: Computed tomography
CTV: Clinical target volume
DLCO: Diffusing capacity of carbon monoxide
FDG-PET: 18F-fluorodeoxyglucose positron emission tomography
FEV1: Forced expiratory volume in one second
FVC: Forced vital capacity
GGO: Ground-grass opacity
GTV: Gross tumor volume
HRCT: High-resolution computed tomography
JCOG: Japan Clinical Oncology Group
NSCLC: Non-small cell lung cancer
NCCN: National comprehensive cancer network
OS: Overall survival
PS: Performance status
PTV: Planning target volume
RBE: Relative biological effectiveness
UICC8: The eighth edition TNM stage classification of malignant tumors
WJOG: West Japan Oncology Group
